# Improving the Thermal and Oxidative Stability of Food-Grade Phycocyanin from *Arthrospira platensis* by Addition of Saccharides and Sugar Alcohols

**DOI:** 10.3390/foods11121752

**Published:** 2022-06-14

**Authors:** Yan Huo, Xiaoyu Hou, Youzhi Yu, Xiaobin Wen, Yi Ding, Yeguang Li, Zhongjie Wang

**Affiliations:** 1 CAS Key Laboratory of Plant Germplasm Enhancement and Specialty Agriculture, Wuhan Botanical Garden, Chinese Academy of Sciences, Wuhan 430074, China; huoyan19@mails.ucas.ac.cn (Y.H.); houxiaoyu17@mails.ucas.ac.cn (X.H.); yuyouzhi15@mails.ucas.ac.cn (Y.Y.); xiaobin@wbgcas.cn (X.W.); dingyi@wbgcas.cn (Y.D.); yeguang@wbgcas.cn (Y.L.); 2 Center of Economic Botany, Core Botanical Gardens, Chinese Academy of Sciences, Wuhan 430074, China; 3 University of Chinese Academy of Sciences, Beijing 100049, China

**Keywords:** phycocyanin, thermal stability, oxidative stability, *Arthrospira platensis*, mannose, mannitol, maltose, glucose

## Abstract

The water-soluble pigment protein phycocyanin (C-PC) from cyanobacteria *Arthrospira* sp. is an excellent natural food colorant and nutritional supplement with a brilliant blue color. However, C-PC is highly unstable, especially at high temperatures and when exposed to oxidative stress. The lack of simple and economical methods for improving the stability of C-PC greatly limits the application of this functional protein in the food industry. This study investigated the effect of adding saccharides (glucose, mannose, galactose, and maltose) and sugar alcohols (mannitol and maltitol) on the stability of food-grade C-PC extracted from *Arthrospira platensis*; the relevant reaction kinetics were also analyzed. The results revealed that glucose, mannose, mannitol, galactose, and maltose could effectively improve the thermal stability of C-PC. This improvement was positively correlated with the concentration of the additives and decreased sharply when the temperature exceeded 60 °C. Furthermore, the results also revealed the instability of C-PC when subjected to oxidative stress and the effectiveness of glucose, mannose, mannitol, and maltose in preventing the oxidative degradation of C-PC. In general, this study demonstrates that glucose, mannose, mannitol, and maltose are promising compounds for promoting the thermal and oxidative stability of C-PC, providing an economical and effective method for C-PC preservation.

## 1. Introduction

C-phycocyanin (C-PC) is a water-soluble pigment-protein complex that is widely distributed in cyanobacteria and functions as a light-energy-capture molecule in the 615–620 nm wavelength range [1]. The monomer of C-PC has a molecular weight of 44 kDa and includes an open-chain linear tetrapyrrole (chromophore of C-PC) and its two covalently linked subunits (α and β peptide chains) [2]. In cyanobacterial cells, this pigment is usually present as a monomer (αβ), trimer (αβ)_3_ and hexamer (αβ)_6_ [3]. C-PC is abundant in cyanobacteria, where its content can even exceed 20% of the dry weight under specific growth conditions (blue light treatment, etc.) [3,4,5]. Owing to its unique brilliant blue color, C-PC is widely used as a natural colorant in multiple industries and has been certified as a safe food additive by the US Food and Drug Administration [6]. The application of C-PC as an ideal blue natural pigment in the food industry has continued to gain attention, with the increasing social interest in health and ‘natural’ appeal [7,8]. Considering the content and proportion of amino acids, anti-inflammatory effects, immunity enhancement, and other health functions [8,9,10] of C-PC, the addition of this pigment to food not only provides a lovely color that consumers find attractive, but also significantly improves the nutritional value of food.

The global C-PC market exceeded USD 110 million in 2018 and is expected to double to more than USD 200 million in the next decade [10]. *Arthrospira* (mainly refers to *Arthrospira platensis*), commercially known as ‘Spirulina’, has a global annual production that currently exceeds 20,000 tons, and is the main biological source of commercial C-PC [4,11]. Although C-PC has significant advantages in terms of color, safety, and biological origins, this algae-derived pigment is characterized by high instability to many extrinsic or intrinsic factors such as temperature, pH, and light. Temperature is one of the main factors affecting the stability of C-PC. The results of previous studies [5,12,13] indicate that the degradation of C-PC is significantly accelerated in the aqueous phase when the ambient temperature exceeds 45 °C, and the degradation rate increases rapidly with increasing temperature. High-temperature processes such as sterilization and cooking are unavoidable in food production; thus, the low stability of the C-PC color to heat greatly limits its application in the food industry.

An increasing number of studies have focused on improving the thermal stability of C-PCs in recent years, and some achievements have been made. The use of additives is the main strategy for stabilizing C-PC, where small-molecule saccharides have received the most attention as potential additives [3,8]. The results of previous studies [7,12,14,15] revealed that high concentrations of certain sugars, including glucose, sucrose, sorbitol, and fructose, could significantly increase the thermal stability of C-PC, where this improvement was positively correlated with the final sugar concentration. Furthermore, sodium chloride, calcium chloride, and polymeric nanofibers have been reported to be effective C-PC-stabilizing agents [5,15]. Processing methods are another strategy for C-PC stabilization, preventing the thermal degradation and color loss of C-PC through crosslinking [16], complexation with polysaccharides (beet pectin) [17], microencapsulation [18], and microcellularization [19]. In general, compared with the complex processing steps and expensive processing methods, the use of additives to improve the thermal stability of C-PC has the advantages of easy operation, high safety of the used additives, and no requirement for expensive instrument, and thus has better application prospects in the food industry [3,8].

As mentioned above, current research has demonstrated the significant effects of saccharide additives on the thermal stability of C-PC in the aqueous phase. However, there are still many areas, including—but not limited to—screening of more sugars that exert thermal protective effects on C-PC, are suitable for food application and have an acceptable price. The reaction kinetics of C-PC under the action of different sugar species and additives need to be further studied for application of these additives to special foods [3,8,20]. In-depth research on the above aspects will deepen our understanding of the mechanisms by which the thermal stability of C-PC is enhanced by sugar and will greatly promote the utility of this excellent natural colorant in the food industry and commercial applications. Notably, the application of C-PC in food is also limited by the oxidizing environment/compounds during food processing and preservation, which is a very important but neglected aspect, and methods of improving the oxidative stability of C-PC need to be studied as a matter of urgency.

The present study systematically evaluates the protective effect of glucose and maltose, which have been reported as efficient saccharides for C-PC stability, as well as four small-molecule additives, including the saccharides mannose and galactose, and sugar alcohols mannitol and maltitol, on the thermal stability of C-PC in aqueous solution at 65 °C. The protective effects and relevant reaction kinetics of these additives at different concentrations are studied. Furthermore, the effects of the six additives on the oxidative stability of C-PC are tested. The objective of this study is to provide a foundation for minimizing the degradation of C-PC for use in the food industry.

## 2. Materials and Methods

### 2.1. C-PC and Additives

C-PC used in this study was extracted from *Arthrospira platensis* cultured in Zarrouk’s medium in the laboratory. The cells were collected by filtration when the culture reached a concentration of *OD_560_* = 1.5–2.0, and then dried by freeze-vacuum drying. The swelling method and ammonium sulfate precipitation were used for the extraction and purification of C-PC. Briefly, 0.05 M phosphate buffer (pH 6.8) was added to *A. platensis* biomass at a ratio of 50:1 (*v*/*w*); the mixture was set aside for 24 h for lysis of the cells by swelling at room temperature. The supernatant containing C-PC was collected by centrifugation at 5000*× g* for 20 min. Subsequently, an equal volume of a precooled saturated ammonium sulfate solution was added to the supernatant at 4 °C and kept for 12 h to precipitate C-PC. The collected C-PC precipitate was dissolved in doubly distilled water (ddH_2_O), and ammonium sulfate was removed using a cellulosic semi-permeable membrane (retained MW: 14,000). Finally, the food-grade C-PC product with a purity of A620/A280 = 1.313 was obtained and stored at −20 °C.

The analytically pure saccharides (glucose, mannose, galactose, maltose) and the sugar alcohols (mannitol and maltitol) were purchased from Sinopharm (Shanghai, China) and used as additives in evaluating the stability of C-PC.

### 2.2. Effects of Additives on Thermal Stability of C-PC

C-PC solutions containing different concentrations of glucose, mannose, mannitol, galactose, maltose, and maltitol were incubated at 65 °C for 60 min to study the effects of these additives on the thermal stability of C-PC. The brief steps of the experiment are as follows: The experimental solution with a C-PC concentration of 0.5 mg mL^−1^ was prepared using 10 mM phosphate buffer (pH 6.8). An 8 mL aliquot of the C-PC solution was placed into a 15 mL centrifuge tube. The additive was weighed and added to the test tube to give final concentrations of 5, 10, 20, 30, and 40%, respectively. The solutions were incubated in a thermostatic water-bath (Amersham MultiTemp Ⅲ, Shanghai, China) for 60 min. The absorbance of the C-PC solutions at 652 nm and 620 nm was measured at 10 min intervals using a spectrophotometer (Shimadzu UV-1800, Suzhou, China). Each experiment was performed in triplicate and a C-PC solution without additives was used as the control.

The C-PC concentration was calculated using the following Equation [12]:

(1) $$C_{PC}\left(\mathrm{mg}\;{\mathrm{mL}}^{-1}\right)=\frac{A_{620}-0.474\times A_{652}}{5.34}$$

In this study, the relative concentration of C-PC (*C_R_*, %) was used to indicate the effect of the additives on the stability of C-PC and was calculated using the following equation:

(2) $$C_{R}=\frac{C_{t}}{C_{0}}\times 100\%$$

where *C_t_* is the concentration of C-PC at time *t* (min) and *C*_0_ is the initial concentration of C-PC.

### 2.3. Kinetic Study of C-PC Degradation

It was confirmed that the degradation of C-PC at high temperatures could be modeled by first-order kinetics [20,21]. The kinetic rate constant (min^−1^) for each sample was calculated using the following equation:

(3) $$\ln\left(\frac{C_{t}}{C_{0}}\right)=-kt$$

where k, *t*, *C_t_*, and *C_0_* are the kinetic rate constant (min^−1^) of degradation, processing time (min), concentration of C-PC at time *t* (min), and the initial concentration of C-PC, respectively. 

The half-life (*t*_1/2_, the time required for the C-PC concentration to decrease to half of the initial value) of each sample was calculated using the equation given below:

(4) $$t_{1/2\;}=\frac{\ln2}{k}$$

### 2.4. Stabilization of C-PC by Additives at Different Temperatures

The effect of the additives on the thermal stability of C-PC at different temperatures was studied using a solution with 0.5 mg mL^−1^ C-PC and a final 30% (*m*/*v*) concentration of each additive. A mass of 2.4 g of glucose, mannose, mannitol, galactose, maltose, or maltitol was added to 8 mL of the C-PC solution, dissolved, and mixed well. Each treatment was performed in triplicate and the solutions were incubated at 40, 50, 60, and 70 °C. The absorbance of the samples was measured at 620 and 652 nm after heat processing for 60 min.

The improvement in the thermal stability of C-PC by the additives was reflected by the relative concentration of C-PC (*C_R_*, %), which was calculated using Equation (2).

### 2.5. Effect of Oxidative Stress on C-PC

Different concentrations of H_2_O_2_ were used to simulate oxidative stress in C-PC. An appropriate amount of 30% H_2_O_2_ (Sinopharm, Shanghai, China) was added to a 0.5 mg mL^−1^ C-PC solution to prepare the test samples with final concentrations of 0.25%, 0.5%, 1.0%, 2.5%, and 4.5% (*m*/*m*) H_2_O_2_, respectively. A C-PC solution without H_2_O_2_ addition was used as a control. Each treatment with different concentrations of H_2_O_2_ was performed in triplicate and the solutions were stored at ambient temperature (20–25 °C). Samples were withdrawn at 24 h and 48 h to measure the absorbance at 620 nm and 652 nm, and the C-PC concentration was calculated using Equation (1).

The relative concentration of C-PC (*C_R_*, %), calculated using Equation (2) was used to evaluate the effects of different oxidative stresses on C-PC degradation.

### 2.6. Effects of Additives on Oxidative Stability of C-PC

The effect of the additives on the oxidative stability of C-PC was evaluated by adding different concentrations of saccharides and sugar alcohols to a solution of C-PC containing 4.5% H_2_O_2_. Glucose, mannose, mannitol, galactose, maltose, and maltitol were added to C-PC/H_2_O_2_ solution to prepare the test samples with 5, 10, 20, 30, and 40% (*m/v*) of additives, respectively. A solution containing 0.5 mg mL^−1^ C-PC and 4.5% H_2_O_2_ was used as a control. After incubation at room temperature for 24 h, the remaining and relative concentrations of C-PC in the experimental samples were calculated using Equations (1) and (2), based on the absorbance values at 620 nm and 652 nm. 

To further clarify the protective effect of the saccharides and sugar alcohols on the oxidative stability C-PC and the related reaction kinetics, the degradation of C-PC with 20% additives and 4.5% H_2_O_2_ was investigated at reaction times of 0, 2, 4, 8, 16, and 24 h. Refer to Section 2.3 for the calculation of the *k* and *t*_1/2_ values.

### 2.7. Statistical Analysis

The relative concentrations of C-PC are presented as the mean ± standard deviation. Significant differences among different treatments were analyzed using SPSS (version 17.0; SPSS, Inc., Chicago, IL, USA) using one-way ANOVA followed by Duncan’s test with a significance level of 0.05.

## 3. Results

### 3.1. Effect of Saccharides and Sugar Alcohols on the Thermal Stability of C-PC

**Figure 1:**
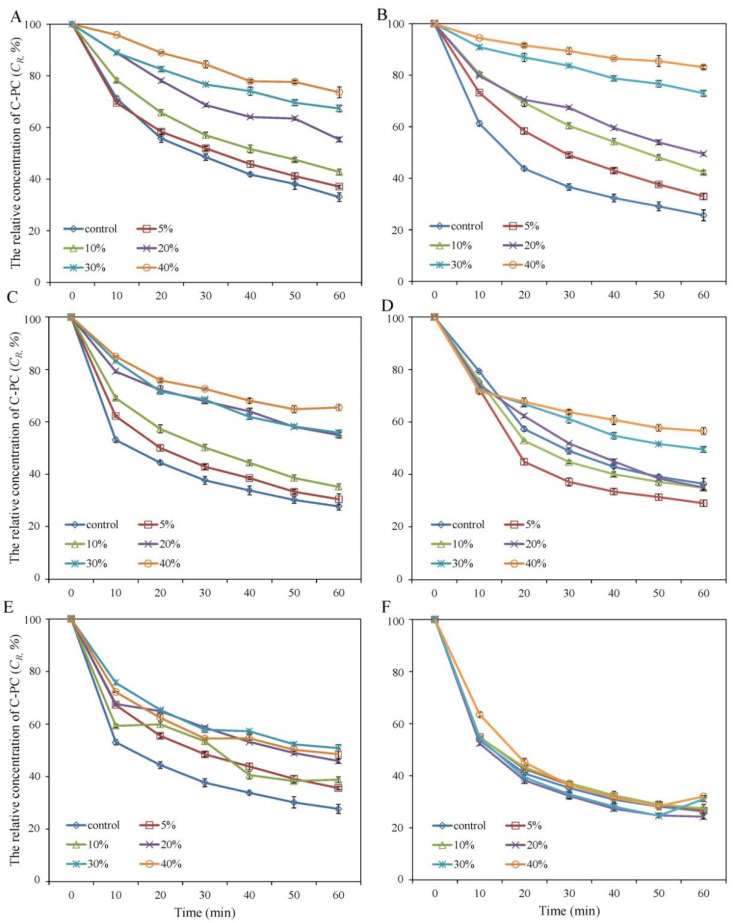
Change in relative concentration of C-PC during incubation with different concentration of additives at 65 °C. (**A**) (glucose); (**B**) (mannose); (**C**) (mannitol); (**D**) (galactose); (**E**) (maltose); (**F**) (maltitol).

The effects of different concentrations of saccharides on the thermal degradation of C-PC at 65 °C were investigated. Figure 1A,B,D,E shows that the addition of 5–40% glucose, mannose, galactose, and maltose significantly reduced the degradation of C-PC. The effect of these additives on improving the thermal stability of C-PC increased with increasing concentration. Up to 73.6 ± 2.1%, 83.1 ± 0.7%, 56.5 ± 1.4%, and 50.8 ± 1.3% of C-PC was preserved after 60 min of heat processing with 40% of the above saccharides, respectively, which is significantly higher (*p* < 0.01) than that achieved in the control without additives (less than 33.1%). 

Similar to the improvement in the thermal stability of C-PC, the addition of mannitol inhibited the thermal degradation of C-PC at 65 °C. As shown in Figure 1C, the relative concentration of C- PC (C*_R_*) was 54.9 ± 0.6%, 55.7 ± 0.5%, and 65.5 ± 1.4% with ascending concentrations of mannitol in the range of 20–40%. These values are significantly higher (*p* < 0.01) than that (27.7 ± 1.3%) of the control. The addition of another sugar alcohol (maltitol) at concentrations of 5–40% did not improve the thermal stability of C-PC (Figure 1F), and there was no significant difference between the treatments and control.

### 3.2. Kinetic Analysis of C-PC Degradation

Two characteristic parameters of the reaction kinetics (the degradation kinetic rate constant (k) and half-life (*t*_1/2_)) of C-PC with different additives at 65 °C were calculated and are listed in Table 1. The k values of C-PC with glucose, mannose, mannitol, galactose, and maltose addition were significantly lower than that of the control and decreased with an increase in the concentration of the additives. C-PC in the solutions with 40% mannose, glucose, and mannitol had the lowest k values of 0.31, 0.51, and 0.71, suggesting that these additives led to the strongest improvement in the thermal stability of C-PC. In contrast with the k value, the *t*_1/2_ was positively correlated with the thermal stability of C-PC. The *t*_1/2_ of C-PC with 40% mannose was 225.20 min, which is significantly longer than 31.14–38.55 min of the control. Among the six additives, maltitol was the only one that caused no significant difference in the k value and *t*_1/2_ relative to the control, which suggests that this sugar alcohol has no effect on the thermal stability of C-PC.

### 3.3. Thermal Stability of C-PC with Additives at Different Temperatures

**Figure 2:**
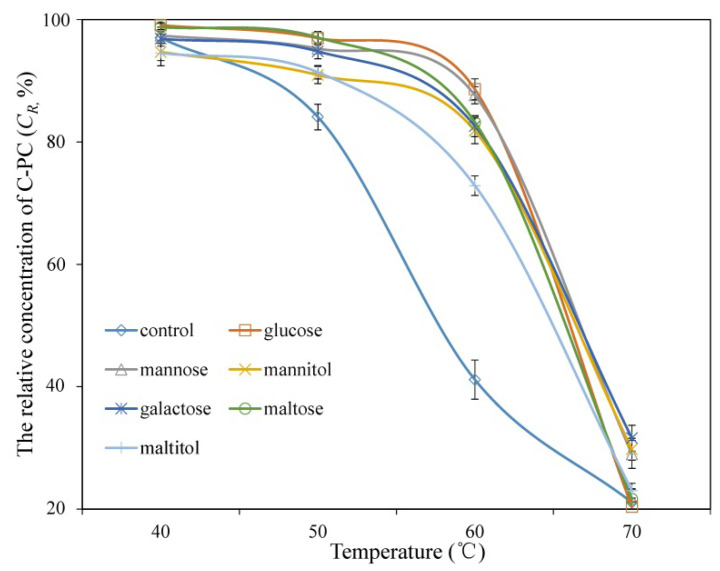
Relative concentration of C-PC treated with 30% additives after incubation for 1 h at different temperatures.

Considering the noticeable improvement in the thermal stability of C-PC at 65 °C upon addition of the saccharides and sugar alcohols, the present study further determined the effect of 30% additives on the thermal stability of C-PC in the temperature range of 40–70 °C over the course of one hour (Figure 2). There was no obvious difference in C*_R_* for the control (without additives) versus the treatments at 40 °C. Thermal degradation of the control increased rapidly in the temperature range of 50–60 °C, whereas the stability of C-PC was strongly improved by the addition of 30% glucose, mannose, mannitol, galactose, maltose, and maltitol, as reflected by the C*_R_* values of 90.9 ± 1.4%–97.1 ± 0.9% at 50 °C and 72.8 ± 1.6%–88.6 ± 2.1% at 60 °C. The effect of the additives on C-PC decreased rapidly when the temperature exceeded 60 °C, and the C*_R_* values of the treated samples were slightly higher than those of the control at 70 °C. Overall, the additives exerted a positive effect on the thermal stability of C-PC, which was strongly correlated with the temperature, and a significant improvement was observed when the temperature was below 70 °C.

### 3.4. Stability of C-PC under Oxidative Stress

**Figure 3:**
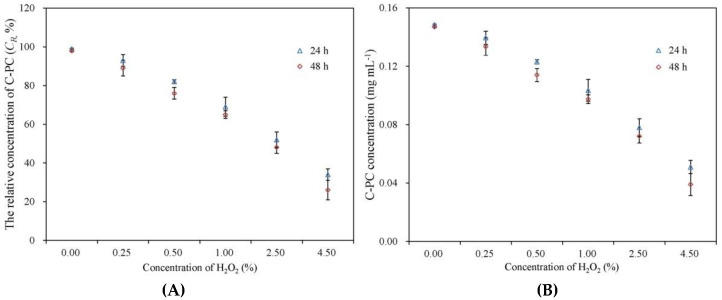
Degradation of C-PC exposed to different concentrations of H_2_O_2_ after 24 and 48 h. (**A**) and (**B**) shows the results of relative concentration of C-PC and C-PC concentration, respectively.

The degradation of C-PC upon exposure to H_2_O_2_ in the concentration range of 0.25–4.5% at room temperature was investigated, as shown in Figure 3. The C*_R_* value was negatively correlated with the concentration of H_2_O_2_. Only 34.2 ± 3.1% and 26.5 ± 4.9% of C-PC were preserved, respectively after 24 h and 48 h incubation with 4.5% H_2_O_2_, whereas little degradation of C-PC was observed in the control. These data confirm that soluble C-PC is sensitive to the oxidative environment. Notably, there was no obvious difference in the C*_R_* at 24 h and 48 h for the same treatment, which indicates that the inactivation of C-PC by H_2_O_2_ mainly occurs in the early stage of incubation.

### 3.5. Improvement in Oxidative Stability of C-PC by Additives

**Figure 4:**
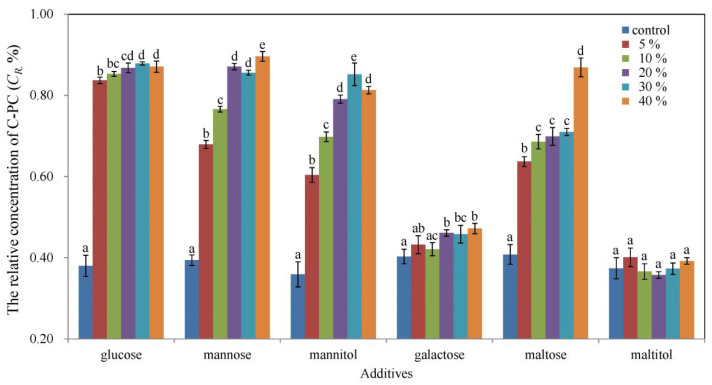
Relative concentration of C-PC treated with 5–40% additives with subsequent exposure to 4.5% H_2_O_2_ for 24 h. Data with different letters indicate significant differences (*p* < 0.05) between the control and treatments.

To explore potential ways to avoid oxidative degradation of C-PC, this study tested the effect of the six additives on the oxidative stability of C-PC under oxidative stress with 4.5% H_2_O_2_ in a 24 h experiment. Glucose, mannose, mannitol, and maltose exerted a positive effect on the oxidative stability of C-PC in the concentration range of 5–40% (Figure 4). The C*_R_* values of the glucose-treated samples were in the range of 83.7 ± 0.8%–87.9 ± 0.4%, with no significant difference for the different concentrations. The positive effects of mannose, mannitol, and maltose on the oxidative stability of C-PC increased with increasing additive concentration, where the highest C*_R_* values were 89.6 ± 1.2%, 85.2 ± 2.8%, and 86.9 ± 2.3%, respectively. No significant improvement was observed with galactose or maltitol addition.

**Figure 5:**
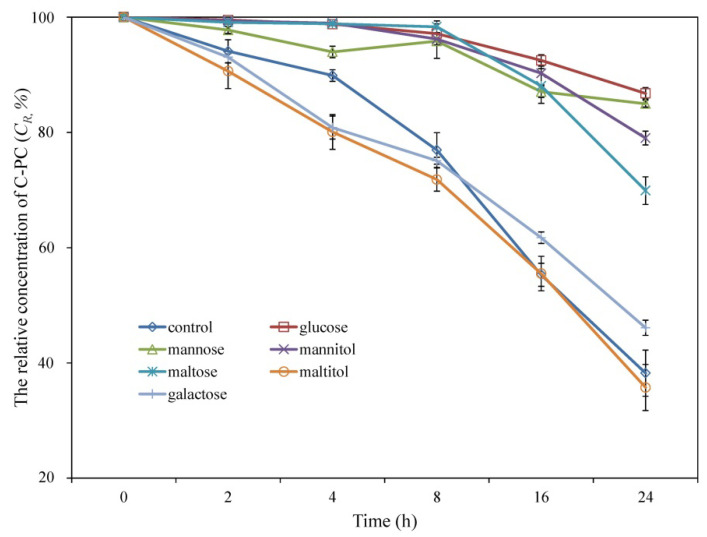
Change in relative concentration of C-PC treated with 20% additives and exposed to 4.5% H_2_O_2_ for 24 h.

Further studies on the degradation of C-PC treated with 20% saccharides and sugar alcohol and exposed to 4.5% H_2_O_2_ yielded similar results (Figure 5). The C*_R_* values of the glucose- and mannose-treated samples at 24 h were 87.3 ± 1.1% and 85.6 ± 0.7%, respectively, and these values are significantly higher than those obtained with mannitol and maltose. The addition of galactose and maltitol did not improve the oxidative stability of C-PC. Table 2 lists the k and *t*_1/2_ values of the C-PC samples with the six additives. The glucose sample had the smallest k (0.09 × 10^−3^ min^−1^) and longest *t*_1/2_ (7.03 × 10^3^ min). The k and *t*_1/2_ values differed by one/two orders of magnitude in the thermal versus oxidative degradation of C-PC, which reflects that the oxidative degradation of C-PC is a relatively slow process.

## 4. Discussion

The high instability of C-PC, especially the degradation and discoloration at high temperatures, has greatly hindered the application and preservation of this excellent natural colorant [3,8,22]. Sugars are considered typical stabilizing compounds that can prevent the degradation of proteins or pigments [14,23]. The effects of these stabilizing agents on the thermal stability of C-PC have attracted extensive attention in the last decade. Previous studies have revealed that glucose, sucrose, sorbitol, and fructose can effectively inhibit thermal degradation and discoloration of C-PC [5,7,12,14,15]. In this study, 48.6 ± 2.1–83.1 ± 0.7% of C-PC was preserved in the samples treated with 40% glucose, mannose, mannitol, galactose, and maltose, respectively, after 60 min incubation at 65 °C, further confirming the positive effect of sugars on improving the thermal stability of C-PC. More importantly, mannose, mannitol, and galactose were demonstrated to be promising compounds for the thermal stability improvement of C-PC for the first time. Glucose, mannose, and mannitol showed better thermal protective effects on C-PC than galactose and maltose. The stabilizing effect of sugars on protein has been proposed to be closely dependent on the N-linked glycosidic bond between the sugar and protein [24,25], water surface tension [12], and increase in the free energy of the system with sugar addition [26]. Few studies on the mechanism of saccharides stabilizing C-PC have yielded inconsistent results. Research by [20] indicated the stabilizing effect of sugars (glucose, sucrose, and fructose) is attributed to the glycosidic bond between sugar and protein, while the results of [12] suggested that increasing water surface tension by adding sugars is the main factor for the improvement of C-PC stability. Recently, the role of water state in C-PC thermostability was confirmed by [7]. Therefore, further studies are urgently needed to unravel the stabilizing mechanism of saccharides on C-PC.

In this study, a positive correlation between the additive content and thermal protective effect on C-PC was revealed by analyzing the relative concentration of C-PC (*C_R_*), kinetic rate constant (k), and half-life (*t*_1/2_). The results are comparable to those in the literature [3,5,12,14,15], and suggest that the prevention of thermal degradation of C-PC largely depends on the concentration of the co-solute saccharides. The *C_R_* value of treatment with 40% mannose, which showed the best protective effect of all the additives in the present study, 83.1 ± 0.7%, is significantly higher than that (32.9 ± 1.1%) achieved with 5% mannose after a 60 min heating process. In previous studies, the feasibility of using the reported high concentration of sugars in the food industry was limited, partially because of the potential negative impact of these sugars on human health. The monosaccharide mannose and sugar alcohol mannitol might be good choices for application in food-grade C-PC because these compounds have little effect on human blood glucose and are suitable for special groups such as diabetics [27]. 

The addition of saccharides and sugar alcohols can effectively inhibit the thermal degradation of C-PC below 65 °C (Figure 1 and Figure 2), which makes it possible to stabilize C-PC during certain heat treatments, such as pasteurization. However, the protective effect of the additives reported in this study and previous literature decreased rapidly when the temperature exceeded 65 °C [7,14,20]. The current findings show that more than 68.5 ± 2.1% of C-PC was degraded after 1 h incubation at 70 °C. Unfortunately, there is still no research on additives that can effectively stabilize C-PC at temperatures above 65 °C. Added salts, such as sodium chloride and calcium chloride [5,15], and processing methods such as microencapsulation [18,28] and microcellularization [19] have proven to be effective for improving the stability of C-PC but are limited by their application effect or cost. The combination of sugar addition and the above methods is a promising approach worthy of special attention in the future. On the other hand, despite some progress in elucidating the mechanisms of thermal degradation and stability enhancement of C-PC, further studies are needed to fully clarify these mechanisms and to provide theoretical support for finding more efficient agents or stabilizing methods [3,8,14,29]. 

In addition to its excellent blue-coloring ability, the antioxidant properties of C-PC and its applications in nutrition and health care have been preliminarily revealed [30]. The tetrapyrrole of C-PC contains unsaturated double bonds, which confer the advantage of antioxidant capacity, but also make it potentially unstable to oxidative stress [30]. However, temperature and pH are considered the main physicochemical factors that affect the stability of C-PC [3,12,31]; in particular, temperature-induced degradation of C-PC and methods for improving the stability have attracted the most attention [5,8,13,20]. The effect of oxidation on the stability of C-PC has not yet been reported. The 24 h and 48 h preservation rates of C-PC were only 34.2 ± 3.1% and 26.5 ± 4.9%, respectively, when exposed to 4.5% H_2_O_2_ (Figure 3), which clearly shows that oxidative stress is a factor that cannot be ignored in the degradation of C-PC. Therefore, improving the oxidative stability must be considered in the processing and preservation of C-PC.

Interestingly, it was revealed that the oxidative stability of C-PC could be improved by adding saccharides and sugar alcohols. Adding 20% glucose, mannose, mannitol, or maltose significantly reduced the kinetic rate constant (k) of oxidative degradation and extended the half-life (*t*_1/2_) of C-PC. This discovery provides a simple and economical method for inhibiting the oxidative degradation of C-PC during processing and storage. Studies have shown that the addition of reducing sugars can increase the stability and antioxidant activity of proteins, and the mechanism is believed to be mainly related to the Maillard reaction between sugars and proteins [32,33,34]. Whether the stabilizing effect of saccharides and sugar alcohols on the oxidative stability of C-PC is the result of the Maillard reaction and the antioxidants produced by this reaction needs further clarification.

## 5. Conclusions

This study demonstrated that addition of glucose, mannose, mannitol, galactose, and maltose can significantly improve the thermal stability of C-PC in the aqueous phase at 65 °C, reflected by the rapid decrease in the kinetic rate constant (k) of degradation and increase in the half-life (*t*_1/2_) of C-PC with an increase in the additive concentration in the range of 5–40%. Mannose has the strongest inhibitory effect on the thermal degradation of C-PC and is revealed herein as an effective C-PC stabilizing compound, along with mannitol and galactose, for the first time. The improvement in the thermal stability of C-PC due to the additives is positively correlated with the additive concentration and varies with temperature. The thermal protective effect of these additives decreased sharply when the temperature exceeded 60 °C. Furthermore, the sensitivity of C-PC to oxidative stress and its degradation were revealed for the first time. More than 50% of C-PC is degraded after 24 h when the concentration of H_2_O_2_ exceeds 2.5%. Oxidative degradation of C-PC is effectively inhibited by the addition of glucose, mannose, mannitol, and maltose. These results suggest that the above additives can not only improve the thermal stability of C-PC, but also protect it from oxidative degradation and are promising compounds for C-PC preservation.

## Figures and Tables

**Table 1:** The kinetic rate constant and half-life values of C-PC at 65 °C with different additives.

Additives	Kinetic Rate Constant Value (*k*) (×10^−2^ min^−1^)					*t*_1/2_ (Half-Life Values) (min)				
	5%	10%	20%	30%	40%	5%	10%	20%	30%	40%
Glucose	1.65	1.42	0.99	0.66	0.51	41.90	48.92	70.26	105.25	135.87
Mannose	1.85	1.43	1.17	0.52	0.31	37.49	48.39	59.05	132.22	225.20
Mannitol	1.98	1.74	1.00	0.97	0.71	34.94	39.74	69.40	71.25	98.12
Galactose	2.06	1.76	1.75	1.17	0.95	33.58	39.48	39.57	59.16	72.96
Maltose	1.72	1.58	1.30	1.13	1.20	40.27	43.97	53.47	61.47	57.57
Maltitol	2.21	2.16	2.36	1.95	1.90	31.39	32.16	29.35	35.56	36.52
Control	1.79–2.21					31.14–38.55				

**Table 2:** The kinetic rate constant and half-life values of C-PC with 20% additives under 4.5% H_2_O_2_ oxidative stress.

Additives (20%)	Kinetic Rate Constant (*k*) Value (×10^−3^ min^−1^)	*t*_1/2_ (Half-Life Values) (×10^3^ min)
Control	0.67	1.04
Glucose	0.09	7.03
Mannose	0.11	6.13
Mannitol	0.16	4.24
Galactose	0.54	2.79
Maltose	0.25	0.97
Maltitol	0.71	1.29

## Data Availability

The data that supports the findings of this study are available within the manuscript.
